# First *in vitro* characterization of three oxytetracycline degradation products against avian pathogenic *Escherichia coli* isolates: antibacterial activity, formation in gizzard, and resistance gene profiling

**DOI:** 10.1128/spectrum.03768-25

**Published:** 2026-07-06

**Authors:** Wassel Zekri, Marie-Lou Gaucher, Maud de Lagarde, William Thériault, David Roy, Alexandra Veress, Francis Beaudry, Mohamed Rhouma

**Affiliations:** 1 Département de Pathologie et Microbiologie, Faculté de médecine vétérinaire, Université de Montréal 70354, Saint-Hyacinthe, Québec, Canada; 2 Groupe de Recherche et d’Enseignement en Salubrité Alimentaire (GRESA), Faculté de médecine vétérinaire, Université de Montréal 70354, Saint-Hyacinthe, Québec, Canada; 3 Centre de Recherche en Infectiologie Porcine et Avicole (CRIPA), Faculté de médecine vétérinaire, Université de Montréal 70354, Saint-Hyacinthe, Québec, Canada; 4 Département de Biomédecine Vétérinaire, Faculté de médecine vétérinaire, Université de Montréal 70354, Saint-Hyacinthe, Québec, Canada; 5 Centre Interdisciplinaire de Recherche sur le Cerveau et l’Apprentissage (CIRCA), Université de Montréal 5622, Montréal, Québec, Canada; Innovations Therapeutiques et Resistances, INTHERES, Université de Toulouse, INRAE, ENVT 551834, Toulouse, France

**Keywords:** antimicrobial resistance, oxytetracycline, degradation products, antibacterial activity, gizzard simulation, APEC

## Abstract

Although oxytetracycline (OTC) has been widely used in poultry since 1950, comprehensive studies characterizing its degradation products for potential therapeutic applications have not been conducted. The aims of the present study were to evaluate the antibacterial activity of three OTC degradation products (4-α-epi-OTC [4-EOTC], α-apo-OTC, and β-apo-OTC) individually and in combination with OTC, to identify and assess the genetic determinants conferring antimicrobial resistance (AMR) in avian pathogenic *Escherichia coli* (APEC) strains exposed to tetracyclines and OTC degradation products, and to assess OTC degradation in a simulated poultry gizzard. The microdilution method was used to determine the minimal inhibitory concentration (MIC) of OTC, chlortetracycline, tetracycline, 4-EOTC, α-apo-OTC, and β-apo-OTC against 15 APEC strains. The fractional inhibitory concentration index (FICI) was calculated to assess the combined effect of OTC with its degradation products. A conjugation assay was conducted to determine whether resistance to OTC and its active degradation products is mediated by the identified tetracycline resistance genes. Results showed that while α-apo-OTC and β-apo-OTC lacked detectable antibacterial activity, 4-EOTC retained inhibitory activity against all tested APEC strains. Notably, a synergistic effect was observed between OTC and 4-EOTC (FICI ≤ 0.5). Preliminary evidence indicated that *E. coli* employed the same resistance determinant (*tetA*) against both OTC and its degradation products, while confirming that OTC remained stable under simulated gizzard conditions. The identification of antibacterial activity in 4-EOTC represents a key finding of this study and warrants further investigation to define its activity spectrum and explore its potential therapeutic applications.

**IMPORTANCE:**

Tetracyclines are among the most extensively used antimicrobials in veterinary medicine, with oxytetracycline (OTC) serving as a cornerstone compound of this family. While several studies have reported that OTC undergoes degradation in the environment, the biological roles of the resulting chemical derivatives in poultry remain poorly understood. The present study demonstrates that one major degradation product, 4-EOTC, retains antibacterial activity against avian pathogenic *Escherichia coli* (APEC) strains and, when combined with OTC, produces a synergistic effect. Importantly, the *tetA* gene confers resistance in APEC against 4-EOTC as it does against OTC, highlighting the possible contribution of OTC degradation products to the spread of antimicrobial resistance (AMR). Furthermore, OTC was found to remain stable under simulated poultry gizzard conditions, indicating that its degradation likely occurs in downstream compartments of the poultry gastrointestinal tract. Collectively, these findings reveal that OTC degradation products are not biologically inert and open new avenues for the development of next-generation tetracyclines.

## INTRODUCTION

Antimicrobial resistance (AMR) is a critical global health issue, posing a serious and growing threat to human, animal, and environmental health worldwide (1). The overuse and misuse of antimicrobials in human and veterinary medicine have been major drivers of the rapid emergence and global dissemination of antimicrobial-resistant bacteria and their resistance determinants (2). The relationship between antimicrobial use in animals and AMR in both animals and humans remains a subject of question for the scientific community and policymakers (3).

According to the report of the World Organization for Animal Health (WOAH) covering the 2020–2022 period, the total quantity of antimicrobial agents intended for use in animals ranged from 70,648 to 74,035 tons, with the tetracyclines and penicillin classes comprising nearly half the agents used across the globe, i.e., 28.29% and 17.75% of the total amount, respectively (4). It is noteworthy that both tetracyclines and penicillins are classified as Veterinary Critically Important Antimicrobial agents in the WOAH List of Antimicrobials of Veterinary Importance (5). Safeguarding the efficacy and longevity of these antimicrobial classes is critical for maintaining animal health and welfare, and ensuring the long-term sustainability of livestock production systems.

**Fig 1:**
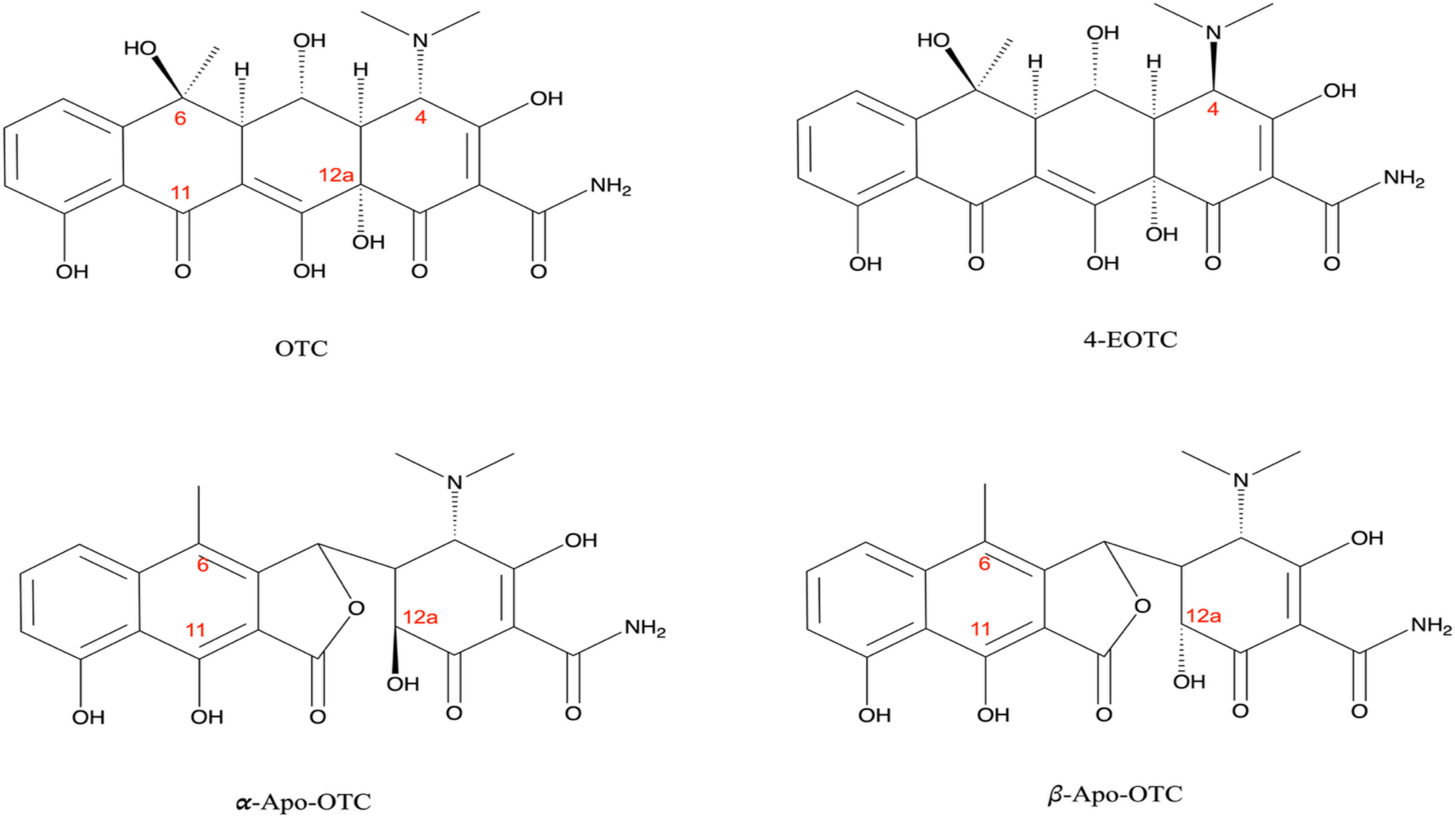
Chemical structures of OTC and its main degradation products, prepared with ChemDraw. OTC, oxytetracycline; 4-EOTC, 4-α-epi-OTC.

In poultry production, tetracyclines, particularly first-generation compounds, such as oxytetracycline (OTC) and chlortetracycline (CTC), are among the most extensively used antimicrobial classes, commonly administered via feed or drinking water for the prevention and treatment of infections caused by *Mycoplasma* spp., *Pasteurella multocida*, and *Escherichia coli* (6–10). For instance, OTC is the only antimicrobial approved in Canada for the treatment of colibacillosis caused by avian pathogenic *E. coli* (APEC) in laying hens (11). This antibiotic is produced by *Streptomyces rimosus*; it was first isolated in Terre Haute, Indiana, USA; hence, the trade name “terramycin” was subsequently assigned to this antibiotic to emphasize its terrestrial origin and natural derivation (12). Oxytetracycline retains the core tetracycline scaffold but is distinguished by an additional hydroxyl substituent at the C5 position (Fig. 1) (13). This antibiotic is a broad-spectrum antibiotic effective against Gram-positive and Gram-negative bacteria (GNB), as well as *Rickettsia*, *Mycoplasma*, and *Chlamydia* species (14). Due to this broad spectrum of activity, safety, and relatively low cost, OTC is widely used in livestock, particularly in poultry production (15).

Following entry via oral, nasal, or cloacal routes, APEC can colonize the gastrointestinal tract of poultry and, under conditions of co-infection with viruses or *Mycoplasma*, or during immunosuppression or stress, invade the mucosal layers and translocate to extra-intestinal sites, subsequently causing multisystemic infections involving organs such as the heart, liver, lungs, spleen, kidneys, reproductive organs, and air sacs (16). In this context, ensuring the oral bioavailability of antimicrobials administered via water or feed for the treatment of colibacillosis in poultry is crucial to reach therapeutic concentrations systemically and in the affected organs. However, OTC is characterized by a low oral bioavailability in poultry, with 30%–90% of the orally administered OTC being excreted in feces, potentially leading to environmental contamination (17).

Several scientific publications have described some OTC’s degradation products (e.g., 4-EOTC, 2-acetyl-2-decarboxy-amidooxytétracycline, α-apo-OTC, and β-apo-OTC) in the environment, mostly in agricultural soils and drainage waters (Fig. 1) (13, 18, 19). Nevertheless, the origin of these degradation products recovered from the environment—whether they are formed in the gastrointestinal tract of poultry or result from microbial or photodegradation of OTC in the environment—remains unknown. It is important to note that the term “metabolites” here refers to chemical products formed when a drug is biotransformed by the body, typically through enzymatic processes, such as oxidation, reduction, or hydrolysis, whereas “degradation products” refers to compounds generated and identified outside the living organism, such as in the environment.

On the other hand, several studies conducted in poultry have reported a very high prevalence of tetracycline resistance in APEC strains, in some cases exceeding 90% of the isolates (20, 21). In GNB, resistance to tetracycline primarily occurs through two mechanisms: efflux pumps and ribosomal protection proteins (22). The most commonly detected resistance genes include *tetA*, *tetB*, *tetD*, *tetE*, and *tetG*, which are predominantly plasmid-encoded (22).

Despite the importance of tetracyclines, particularly OTC, in the poultry sector—notably in laying hens—very few studies have characterized the formation of OTC metabolites in poultry, their stability compared to the parent compound, their antibacterial activity, and the bacterial resistance mechanisms to these products relative to the parent compound.

Therefore, the present study aimed to address three main objectives: (i) to assess the antibacterial activity of three OTC degradation products against 15 APEC strains, in comparison with the parent compound and other tetracycline compounds (tetracyclines [TC], CTC), tested both individually and in combination with OTC; (ii) to determine whether resistance to OTC and its active degradation products is mediated by the same tetracycline resistance genes; and (iii) to investigate, using simulated *in vitro* gizzard conditions, whether the extreme physicochemical environment of the proximal gastrointestinal tract in poultry promotes OTC degradation.

## MATERIALS AND METHODS

### Chemical products and reagents

The three major degradation products of OTC and commercially available: 4-EOTC (purity 95.0%), α-apo-OTC (purity 90.0%), and β-apo-OTC (purity >85.0%) were purchased from Toronto Research Chemicals Inc. (Vaughan, ON, Canada) and used in this study. OTC (purity >94.0%), CTC (purity >90.0%), TC (purity >85.0%), and nalidixic acid (Nal) (purity >98.0%) were purchased from Sigma-Aldrich (St. Louis, MO, USA). All solvents used were of Optima liquid chromatography (LC) grade (Fisher Scientific, Whitby, ON, Canada), and Optima LC grade H_2_O was used for dilutions. Pepsin and acetonitrile, used to assess the stability of OTC under simulated poultry gizzard conditions, were purchased from Sigma-Aldrich (St. Louis, MO, USA).

### Bacterial strains

To evaluate the antibacterial activity of the different chemical compounds (TC, CTC, OTC, and OTC degradation products), 15 APEC strains were used (Table 1). These strains were isolated from laying hens diagnosed with colibacillosis. They were predicted to be APEC based on the presence of the five major virulence-associated genes commonly found in highly pathogenic APEC strains, as described by Johnson T et al. (23). All isolates were obtained from the WOAH Reference Laboratory for *E. coli* (EcL) at the Université de Montréal. Whole-genome sequencing (WGS) of all 15 APEC strains was performed using the Illumina technology. A total of 14 isolates were sequenced using the iSeq platform (Illumina, San Diego, CA) with a 2 × 151 bp paired-end protocol, and one isolate was sequenced using the MiSeq platform (Illumina, San Diego, CA) with a 2 × 300 bp paired-end protocol. Antimicrobial resistance genes in the different APEC strains were identified using the ResFinder tool (version 4.6.0) available on the Center for Genomic Epidemiology platform (https://genepi.dk/). The analysis was performed in September–October 2024 using default parameters, except for the species setting, which was restricted to *E. coli*. These analyses were conducted to determine the presence of *tet* genes within these strains.

**TABLE 1:** Minimal inhibitory concentrations (MICs) of tetracyclines (TC, CTC, and OTC) and some OTC degradation products against 15 APEC strains determined by microdilution method^*a*^

APEC strains	*tet* genes	MIC (CTC)	MIC (TC)	MIC (OTC)	MIC (4-EOTC)	MIC (α-apo-OTC)	MIC (β-apo-OTC)
ECL15813	*tetA*	64	256	512	>256	>256	>256
ECL25364	*tetB*	128	256	512	>256	>256	>256
ECL20552	*tetA*	32	256	256	>256	>256	>256
ECL24206	*tetA*	32	128	256	>256	>256	>256
ECL25618	*tetA*	32	256	256	>256	>256	>256
ECL24026	*tetA*	64	256	256	>256	>256	>256
ECL21752	*tetA*	64	256	256	>256	>256	>256
ECL23871	Absent	4	4	2	64	>256	>256
ECL21813	Absent	8	8	4	64	>256	>256
ECL22860	Absent	4	4	2	32	>256	>256
ECL19808	Absent	4	8	4	32	>256	>256
ECL20423	Absent	1	1	1	16	>256	>256
ECL25149	Absent	4	2	1	64	>256	>256
ECL20719	Absent	8	4	1	64	>256	>256
ECL23136	Absent	4	1	1	64	>256	>256

^*a*^ OTC, oxytetracycline; CTC, chlortetracycline; TC, tetracycline; 4-EOTC, 4-α-epi-OTC; MIC, minimal inhibitory concentration. All assays were performed in triplicate.

In addition, the phenotypic resistance profile of these strains was determined for the different compounds by measuring the minimal inhibitory concentration (MIC) using the microdilution method (24–26). The correlation between the genotypic and phenotypic profiles, as well as cross-resistance among the different compounds, was also assessed using this same method.

The laboratory strain Nal-resistant *E. coli* K-12 was used as the recipient bacterium in conjugation experiments. The MICs of OTC and its degradation products were determined for this strain using the microdilution method as described below.

### Assessment of the antibacterial activity of the selected chemical compounds

#### Antibacterial activity of chemical compounds tested individually

To characterize the antibacterial activity of the various chemical compounds selected (OTC, TC, CTC, 4-EOTC, α-apo-OTC, and β-apo-OTC) against the different APEC strains, the broth microdilution method was performed in triplicate following the guidelines of the Clinical and Laboratory Standards Institute (CLSI), as previously described (24, 25). The reported MIC values correspond to results obtained in at least two independent assays. In fact, the antimicrobial activity assay was performed in sterile 96-well polystyrene microplates (Cat. #82.1582001). A volume of 100 μL of fresh Mueller–Hinton broth (Fisher Scientific, Le Pont de Claix, France) was added to each well. Sterile water was used to dissolve the various chemical compounds (OTC, TC, CTC, 4-EOTC, α-apo-OTC, and β-apo-OTC), and 100 μL of each prepared solution was dispensed into the twelfth row of the microplate, which already contained 100 μL of Mueller–Hinton broth. The initial concentrations were either 256 or 512 µg/mL, depending on the previously determined AMR profile of each bacterial strain. Serial twofold dilutions were then performed from the twelfth to the third row, resulting in final antibiotic concentrations ranging from 0.5 to 256 µg/mL or from 1 to 512 µg/mL. Bacterial inocula were prepared from overnight cultures of each APEC strain and were diluted in sterile saline solution (0.85%) standardized to 0.5 McFarland. From this bacterial suspension, 100 µL was taken and dissolved in 9.9 mL of Mueller–Hinton broth to prepare a bacterial solution with an approximate concentration of 5 × 10^5^ CFU/mL, and 100 µL of that was further added to each well. Two rows without chemical compounds served as controls: one positive control consisted of 100 µL of the bacterial solution with 100 µL of Mueller–Hinton broth, and one negative control consisted of 200 µL of Mueller–Hinton broth without bacterial inoculum. The microplates were then incubated at 37°C for 18 to 24 h, and the MIC was determined as the lowest concentration that resulted in the inhibition of visual bacterial growth (no visible turbidity was observed after incubation). According to CLSI guidelines, *E. coli* strains with a tetracycline MIC ≥16 µg/mL were classified as resistant (R), those with an MIC of 8 µg/mL as intermediate (I), and those with an MIC ≤4 µg/mL as susceptible (S) (27).

#### Antibacterial activity of OTC in combination with its degradation products

The impact of each OTC degradation product (4-EOTC, α-apo-OTC, or β-apo-OTC) on the MIC of OTC was assessed in triplicate using the broth microdilution method, while maintaining the same conditions as described in section “Antibacterial activity of chemical compounds tested individually.” This evaluation aimed to determine whether the combination with the degradation products enhanced, reduced, or had no effect on the antibacterial activity of the parent compound (OTC). Indeed, twofold serial dilutions of OTC and each of its three degradation products were prepared separately in 1 mL dilution tubes. A total of 64 combinations were generated by dispensing the eight serial dilutions of OTC along the horizontal rows of a microplate and the eight serial dilutions of a given OTC degradation product along the vertical columns, as previously described (28). This design ensured that each OTC concentration was tested against eight twofold concentrations of the corresponding degradation product.

After the bacterial suspension was added, the highest concentration of each component in the combination was set to match the MIC of OTC observed for resistant strains and the MIC of 4-EOTC observed for susceptible strains.

To characterize the antibacterial effect of the combination of OTC and its three-degradation products for the 15 APEC strains, the fractional inhibitory concentration index (FICI) was calculated as the sum of the fractional inhibitory concentration (FIC) of OTC [FIC (OTC)] and that of the fractional inhibitory concentration of the OTC degradation products [FIC (degradation product)] using the following equation:

FICI = FIC (OTC) + FIC (OTC degradation product)

With


$$FIC\;(OTC)=\frac{MIC(OTC)\;in\;presence\;of\;an\;OTC\;degradation\;product}{MIC(OTC)}$$

And


$$FIC\;(OTC\;degradation\;product)=\frac{\text{MIC(OTC degradation product) in presence of OTC}}{\text{MIC(OTC degradation product)}}$$

After calculation, the FICI was interpreted as previously described (29), to determine the effect of the combination: FICI ≤0.5 indicated synergy, 0.5 < FICI ≤ 0.75 indicated partial synergy, 0.75 < FICI ≤ 1 indicated an additive effect, 1 < FICI ≤ 4 indicated a neutral effect, and FICI >4 indicated antagonism.

### Conjugation assays

The MOB-suite was used to identify plasmid-derived contigs in the seven tetracycline-resistant APEC strains. Following confirmation of the presence of *tet* genes on these contigs, putative plasmids were reconstructed *in silico* using the MOB-recon module, as previously described (30). The reconstructed plasmids were then characterized based on plasmid clusters, nearest replicon types, and MOB types. Finally, their mobility potential was predicted, as outlined previously (30).

Three tetracycline-resistant APEC strains were selected for the conjugation assay. These strains were used as donor bacteria. Another *E. coli* K-12 strain resistant to Nal was used as the recipient. The susceptibility of the recipient strain to OTC and of the donor strain to Nal was verified prior to the assay. Strains were cultivated separately in Luria Bertani (LB) broth (EMD Chemicals, Darmstadt, Germany) with the appropriate antibiotic (30 µg/mL OTC or 40 µg/mL Nal). After 18 to 24 h of incubation at 37°C, 100 µL of the donor strain was mixed with 100 µL of the recipient strain in a microtube and centrifuged. The supernatant was discarded, and the solution was resuspended by adding 10 µL of LB broth without antibiotics. Then, the mixture was incubated in LB agar (EMD Chemicals, Germany) for 5 h at 37°C to allow conjugation. After incubation, bacteria were collected by washing the agar with 1 mL of LB broth. Finally, 100 µL were inoculated on LB agar containing OTC (30 µg/mL) and Nal (40 µg/mL) to isolate transconjugant strains (31).

The MICs of the recipient strain prior to conjugation and of the transconjugant strain for OTC and 4-EOTC were determined as described in section “Antibacterial activity of chemical compounds tested individually.”

After MIC determination, a PCR assay was performed to confirm that the transconjugant strain corresponded to the recipient that had acquired the OTC resistance gene, rather than the donor strain acquiring resistance to Nal during conjugation. This PCR assay was conducted using the conditions previously described by Iguchi A et al. (32), and targeted the *wzy* gene encoding the O-antigen polymerase, associated with the O25 serogroup (which was absent from the recipient strain).

### Simulated gizzard conditions and analytic method

The simulated gizzard conditions were used to assess whether OTC undergoes degradation during passage through the gizzard, and if so, whether the resulting products retain antibacterial activity against the various APEC strains used in this study. The composition of the simulated gizzard was prepared as previously described (33). Briefly, in 50 mL tubes, 12 mL of sterile water was added to 1 M sodium chloride (NaCl) (Fisher Scientific, Ottawa, ON, Canada) and 10 g/L pepsin. The pH was adjusted to 2 using hydrochloric acid (HCl) (Fisher Scientific, Ottawa, ON, Canada). The tubes were then incubated in a thermostatically controlled oven at 42°C for 15–20 min (33). Then, a total of 40 mg of OTC, dissolved in 1 mL of water, was added to the tube to assess OTC degradation under simulated poultry gizzard conditions. This OTC dose corresponds to the average amount administered *in vivo* to a 2-kg body weight laying hen. The tube was incubated at 42°C with agitation at 350 rpm on a shaker. Samples were collected from the simulation at 0, 5, 10, 15, 30, 45, 60, and 120 min after OTC addition to the gizzard simulation. At each time point, 333 µL of the solution was transferred into a microtube containing two volumes of acetonitrile to stop enzymatic activity. The mixtures were then centrifuged at 12,000 × *g* for 5 min, and 500 µL of the resulting supernatant was collected for acetonitrile evaporation, as previously described. Two control conditions were included in this experiment: (i) a simulation without OTC and (ii) a simulation without pepsin. These controls were designed to assess the intrinsic antibacterial activity of the gizzard simulation matrix in the absence of OTC, and to evaluate OTC degradation in the absence of pepsin, primarily due to the chemical activity of HCl. The MIC of OTC in the gizzard simulation was determined at each time point against the 15 APEC strains as described in section “Antibacterial activity of chemical compounds tested individually.” All assays were performed in triplicate.

Simultaneously, the concentrations of OTC and its degradation products were determined at each time point using a liquid chromatography coupled with tandem mass spectrometry (HPLC-MS/MS) method based on previous work (34). Briefly, from each sampling time (0, 5, 10, 15, 30, 45, 60, and 120 min), 2 μL were injected into a nano-flow Thermo Scientific Vanquish Neo UHPLC system (San Jose, CA, USA) configured for pre-concentration using a Thermo Scientific PepMap Neo 5 µm C18 300 µm × 5 mm trap cartridge in back-flush mode. Rapid sample loading was performed at a flow rate of 20 μL/min on the trap cartridge. OTC, metabolites, and CTC (i.e., internal standard) were separated using a mobile phase consisting of 0.1% formic acid in water (A) and 0.1% formic acid in an 80% acetonitrile and 20% water mixture (B) with a 12-min gradient from 5% to 90% B at a flow rate of 400 nL/min. The column wash (i.e., ZebraWash) was performed and reconditioned to the initial mobile phase condition. The separation was conducted on a Thermo Scientific Neo C18 2 µm × 75 µm × 150 mm nano column, which was coupled to a Thermo Scientific Q Exactive Plus hybrid quadrupole-Orbitrap mass spectrometer with a Thermo Nanospray Flex ion source. The Nanospray electrode was set to 2,200 V, and the ion transfer tube temperature was maintained at 200°C. MS detection was performed in positive ion mode and operating in scan mode at high resolution and accurate mass. The scan range was set to *m/z* 200–600. Data was acquired at a resolving power of 70,000 (FWHM) using an automatic gain control target of 1.0 × 10^6^ and a maximum ion injection time of 200 ms. Targeted drug quantification was performed at the MS^1^ level using specific precursor masses based on the monoisotopic masses (i.e., [M+H]^+^ ions). Quantification was performed by extracting specific precursor ions using a 5 ppm mass window. Instrument calibration was performed prior to all analysis, and mass accuracy was notably below 1 ppm using Thermo Pierce calibration solution and automated instrument protocol. OTC and metabolite quantification were performed using the peak-area ratio of OTC or metabolites and the internal standard, CTC, and concentrations were determined by interpolating unknowns from the calibration curve constructed with standards. The observed precision and accuracy were <15%.

### Statistical analysis

The MIC values of TC, CTC, and OTC against the resistant APEC strains were compared using the Kruskal–Wallis rank-sum test, followed by pairwise Wilcoxon tests to assess overall differences between the molecules and to perform multiple comparisons, respectively. The *P* values were adjusted using the Benjamini–Hochberg method to control the false discovery rate. All analyses were conducted in R (version 4.5.1). The *kruskal.test*() and *pairwise.wilcox.test*() functions (base R) were applied to evaluate overall and pairwise differences, respectively, with statistical significance defined as *P* < 0.05.

## RESULTS

### Genotypic characterization of tetracycline resistance in APEC strains

In the present study, tetracycline resistance genes were detected in seven of the 15 APEC strains analyzed by WGS. Among these, six strains carried the *tetA* gene and one strain carried the *tetB* gene (Table 1). No *tet* gene was detected in the remaining eight strains. The presence of these genes underlies the phenotypic resistance to tetracyclines observed in the seven APEC strains (Table 1).

### Phenotypic susceptibility of APEC strains to tetracyclines (TC, OTC, CTC) and OTC degradation products

The MICs of TC, CTC, OTC, and OTC degradation products were determined against the 15 APEC strains included in this study (Table 1). For TC, CTC, and OTC, results showed that the presence of *tetA* or *tetB* gene was associated with an MIC greater than the tetracycline breakpoint (16 µg/mL) (Table 1). For the seven resistant APEC strains, the MICs of OTC and TC were significantly higher compared to CTC (*P* = 0.0024). No significant difference was observed between MIC (OTC) and MIC (TC) against the same resistant APEC strains (*P* = 0.1088). Nevertheless, the MICs of the three tetracyclines included in this study for the sensitive strains were equal to or less than 8 µg/mL (Table 1).

For OTC degradation products, all MICs were higher than the MIC of the parent compound in the 15 APEC strains. The MICs of α-apo-OTC and β-apo-OTC were consistently greater than 256 µg/mL for both susceptible and resistant APEC strains. All MICs of 4-EOTC against the 15 APEC strains were ≥16 µg/mL, with susceptible APEC strains showing lower MICs than resistant ones (Table 1). Interestingly, one APEC strain (ECL20423) exhibited a 4-EOTC MIC of 16 µg/mL, corresponding to the established breakpoint, whereas the MICs for the other susceptible strains were two- to fourfold higher than this threshold.

### Antibacterial effects of combining OTC with its degradation products

**Fig 2:**
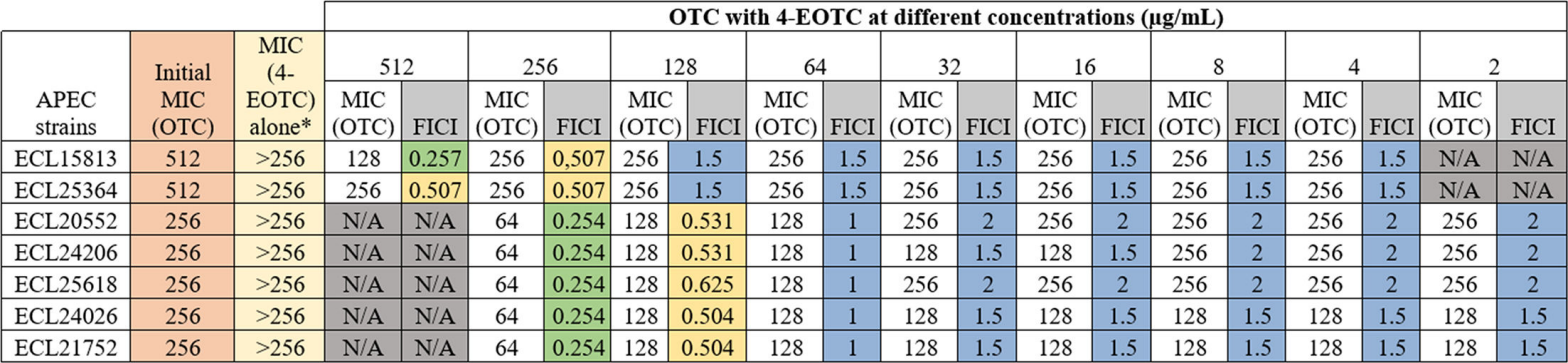
Combinatory effect of OTC and 4-EOTC against tetracycline-resistant APEC strains (*n* = 7) determined by the checkerboard method. OTC, oxytetracycline; CTC, chlortetracycline; TC, tetracycline; 4-EOTC, 4-α-epi-OTC; FICI, fractional inhibitory concentration index; MIC, minimal inhibitory concentration; N/A, not applicable (the assay was not performed at this concentration), green highlight synergistic effect (FICI < 0.5), yellow highlight partial synergistic effect (0.5 < FICI < 0.75), and blue highlight neutral effect (1 < FICI < 4). All assays were conducted in triplicate. *For these strains, the MIC of 4-EOTC was considered to be 512.

The FICI was calculated to characterize the antibacterial effect of the combination of OTC and its three degradation products against the 15 APEC strains. First, the FICI was determined for the seven tetracycline-resistant APEC strains to evaluate whether this combination could enhance the antibacterial activity of OTC, potentially transforming an ineffective compound alone into an effective combination. In fact, the addition of different concentrations of α-apo-OTC or β-apo-OTC to OTC in the microplate in the broth microdilution assay did not affect the initial MIC of OTC for any of the tested APEC strains (Tables S1 and S2). Even a high concentration (256 µg/mL) of these two degradation products did not reduce the initial MIC of OTC. However, the addition of certain concentrations of 4-EOTC to OTC in the microplate was able to reduce the initial MIC of OTC for the seven tetracycline-resistant APEC strains (Fig. 2). For these resistant strains, the MIC of 4-EOTC was assumed to be 512 µg/mL for the calculation of the FICI of the OTC and 4-EOTC combination. Indeed, the value of FICI of this combination was <0.5 when the 4-EOTC concentration was equal to the initial MIC (OTC) for six out of the seven tetracycline-resistant APEC strains, indicating a synergistic effect for these strains (Fig. 2). The APEC strain ECL25364 for which the 4-EOTC–OTC combination did not demonstrate synergistic activity was the only one among the seven tetracycline-resistant APEC strains found to harbor the *tetB* gene (Table 1). When the 4-EOTC concentration was reduced to ½ initial MIC (OTC), the effect of this combination was qualified as partial synergistic for the seven tetracycline-resistant APEC strains (Fig. 2). Finally, the FICI was 1, 1.5, or 2 when 4-EOTC was present with less concentration [<½ initial MIC (OTC)], indicating a neutral effect of this combination (Fig. 2).

**Fig 3:**
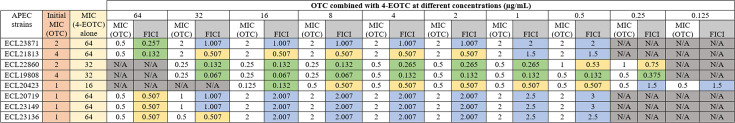
Combinatory effect of OTC and 4-EOTC against tetracycline-susceptible APEC strains (*n* = 8) determined by the checkerboard method. OTC, oxytetracycline; 4-EOTC, 4-α-epi-OTC; FICI, fractional inhibitory concentration index; MIC, minimal inhibitory concentration; N/A, not applicable (the assay was not performed at this concentration), green highlight synergistic effect (FICI < 0.5), yellow highlight partial synergistic effect (0.5 < FICI < 0.75), and blue highlight neutral effect (1 < FICI < 4). All assays were conducted in triplicate.

Second, the FICI was calculated for the eight tetracycline-susceptible APEC strains, showing that in the presence of 4-EOTC, even at lower concentrations, its value remained <0.75 for some strains (ECL22860 and ECL19808), thereby qualifying the combination as synergistic or partially synergistic (Fig. 3). Nevertheless, with the rest of the strains, the combination was qualified as synergistic or partially synergistic when 4-EOTC was present with concentrations near to MIC (4-EOTC) and neutral when 4-EOTC concentrations were lower (Fig. 3).

### Susceptibility of the transconjugant strain to OTC and 4-EOTC

The *tet* genes identified in the seven tetracycline-resistant APEC strains were predominantly plasmid-borne, with only one strain carrying a chromosomal *tet* gene, as predicted using MOB-suite (Table S3). These predicted plasmids were associated with three distinct replicon types. The characterization of the reconstructed plasmids is presented in Table S3. Based on these findings, three representative tetracycline-resistant APEC strains: ECL15813, ECL25618, and ECL25364, each harboring one of the three different replicon types and predicted as conjugative, were selected for conjugation assays. Conjugative transfer was successfully achieved for only one of the tested strains, ECL15813, which harbored the *tetA* gene.

The MICs of OTC and 4-EOTC were determined, using the microdilution method, for the recipient bacteria (*E. coli* K-12 strain). The MIC of OTC alone was 1 µg/mL, while that of 4-EOTC was 16 µg/mL for this strain. For the transconjugant strain, the MIC of OTC was 512 µg/mL, and that of 4-EOTC exceeded 256 µg/mL. These MICs were equal to those of the donor strain (Table 1, ECL15813) and higher than the MICs of the recipient strain.

Following the susceptibility testing, a PCR assay targeting the *wzy* gene was conducted to verify its absence in the transconjugant strain. The negative result obtained in the present study confirms that the transconjugant strain corresponded to the recipient that had acquired the OTC resistance gene.

### Degradation of OTC under simulated poultry gizzard conditions

**Fig 4:**
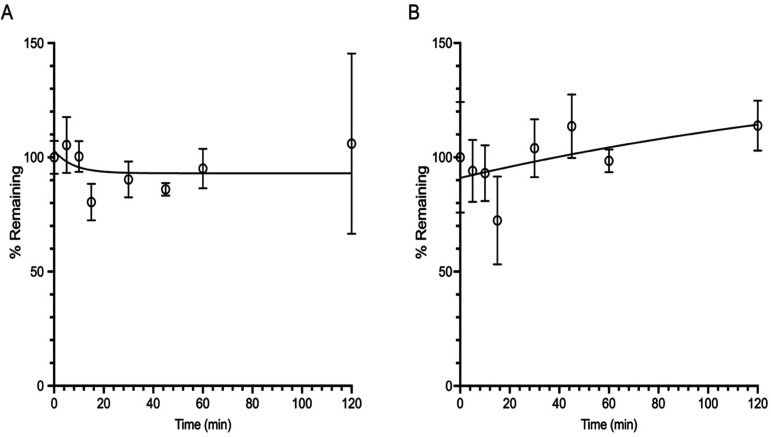
The remaining OTC in the gizzard simulation until 120 min of incubation at 42°C (**A**: in the absence of pepsin; **B**: in the presence of pepsin).

OTC concentrations in the gizzard simulation were monitored by HPLC-MS/MS, with samples collected at 0, 5, 10, 15, 30, 45, 60, and 120 min after OTC addition. No evidence of OTC transformation or degradation was observed at any time point, regardless of pepsin presence (Fig. 4). Furthermore, no OTC degradation products were detected throughout the experiment.

### Antibacterial activity of OTC under simulated poultry gizzard conditions

At each sampling time (0, 5, 10, 15, 30, 45, 60, and 120 min after OTC addition), MICs of the gizzard simulation against all APEC strains were determined using the microdilution method. No differences in MICs were observed between simulations with or without pepsin across all time points. For the eight tetracycline-susceptible APEC strains, MICs ranged from 0.97 to 7.8 µg/mL, closely matching the initial MIC of OTC. Similarly, the seven tetracycline-resistant strains showed MIC values of 312.5 or 625 µg/mL.

## DISCUSSION

To the best of our knowledge, this is the first comprehensive study to investigate the antibacterial activity of three OTC degradation products, the AMR mechanisms employed by some clinical APEC strains against these products, and the potential formation of these chemical compounds in the poultry proximal gastrointestinal tract under simulated gizzard conditions. First, our study confirms the excellent correlation (100%) between the predicted genotypic resistance using WGS and the phenotypic resistance profile observed through microdilution tests. Indeed, the presence of either the *tetA* or *tetB* gene in the seven APEC strains was associated with phenotypic resistance to TC, CTC, and OTC. This result corroborates previous studies that have demonstrated cross-resistance in *E. coli* strains to different compounds within the first-generation tetracycline family (10, 35, 36).

On the other hand, in the present study, MICs of OTC and TC were significantly higher in tetracycline-resistant APEC strains compared to those of CTC (Table 1). This finding is consistent with the study of Sengeløv et al., who reported that in tetracycline-resistant *E. coli* strains isolated from pigs and broilers, the MICs of OTC and TC were consistently >32 µg/mL, whereas the MICs of CTC ranged from 8 to >32 µg/mL (37). These results indicate heterogeneity in the efficacy of the different compounds within this antimicrobial class (10).

Although the tetracycline class has been available on the market since the 1940s—specifically CTC, which was introduced in 1948, and OTC, making its appearance in the market in 1951, both derived from *Streptomyces* spp. (38), surprisingly very few studies have characterized the antibacterial activity of their degradation products (37). In the present study, we characterized the antibacterial activity of three OTC degradation products that have previously been described in the environment and are also commercially available. This selection was further justified by the fact that OTC is the only antimicrobial currently approved for the treatment of colibacillosis in laying hens in Canada (11). Moreover, this choice aligns with the origin of the APEC isolates used in this study, which were obtained from necropsied laying hens diagnosed with colibacillosis. In the current study, results from the microdilution assays confirmed that neither α-apo-OTC nor β-apo-OTC displayed antibacterial activity against the 15 APEC strains evaluated. However, the 4-EOTC retained antibacterial activity, especially against tetracycline-sensitive APEC strains, although its efficacy remained lower than that of the parent compound (OTC). To the best of our knowledge, this finding is consistent with the only study in the scientific literature by Sengeløv et al., which examined the antibacterial activity of certain OTC degradation products using the same microdilution method against *E. coli* strains of animal origin (pigs and broilers) (37). An important distinction between our study and that of Sengeløv et al. is the concentration range tested. Indeed, we evaluated OTC degradation products at substantially higher concentrations (256–512 µg/mL), whereas concentrations used in this previous study were limited to 32 µg/mL. We therefore confirmed that even at these elevated concentrations, α-apo-OTC and β-apo-OTC exhibited no antibacterial activity, whereas 4-EOTC retained antibacterial activity against APEC strains. Similarly, Sorensen et al. confirmed this observation when they evaluated the antibacterial activity of OTC and three of its degradation products (4-EOTC, α-apo-OTC, and β-apo-OTC) using the microdilution method against environmentally relevant bacteria (*Pseudomonas*, *Agrobacterium*, *Moraxella*, *Bacillus*, and *E. coli*). They reported that the MIC of OTC was lower than that of 4-EOTC (1 vs 16 µg/mL against *Pseudomonas* and 0.25 vs 1 µg/mL against *Agrobacterium*, *Moraxella*, and *Bacillus*), whereas α-apo-OTC and β-apo-OTC exhibited very weak antibacterial activity, with MICs ≥ 32 µg/mL (39). The authors suggested that these two OTC degradation products have a very narrow spectrum of activity compared to OTC (39). To explain the low antibacterial activity of the three OTC degradation products compared to the parent compound observed in this study, it can be hypothesized that this would primarily be attributed to chemical modifications occurring during their formation. Indeed, the antibacterial activity of tetracyclines requires an intact linear DCBA naphthacene ring system, with a C1–C3 diketo substructure on the A-ring and an exocyclic C2 carbonyl or amide group (Fig. 1) (40). In contrast, α-apo-OTC and β-apo-OTC are formed through the elimination of H_2_O involving the hydrogen at position C-5a and the hydroxyl group at position C-6, coupled with cleavage of the B-ring to stabilize the molecule (Fig. 1) (41). Consequently, the linear DCBA naphthacene ring system is disrupted in these two chemical products, which explains the absence or extremely low antibacterial activity of α-apo-OTC and β-apo-OTC observed in our study, as well as in previous reports (37, 39). According to Lykkeberg et al., the antibacterial activity of these two degradation products is estimated to be only 7%–10% of that of the parent compound, OTC (41). However, in our study, we confirmed that 4-EOTC retains antibacterial activity, which could be explained by the fact that its chemical structure preserves the linear DCBA naphthacene ring system (Fig. 1). In addition, 4-EOTC is the 4R isomer of OTC, and this isomerization explains the reduction of its antibacterial activity, knowing that the natural 4S isomer of the C4-dimethylamine group is necessary for optimal antibacterial activity, while epimerization to its 4R isomer decreases this activity against GNB (40). According to Wang et al., 4-EOTC has 30% of the antibacterial activity compared to that of the parent compound, OTC (42).

To the best of our knowledge, the present study is the first to use the FICI to characterize the effect of combining OTC with three of its degradation products. Indeed, the FICI is commonly employed to evaluate interactions between antimicrobial agents (43), between antimicrobials and dicarboxylic acid (44), between antimicrobial and antimicrobial peptide (45), and between antimicrobials and essential oils (46). In addition, this index was chosen according to the context of the current study as it is considered the standard reference parameter to quantify pairwise drug interactions in antimicrobial research (47, 48). For the resistant APEC strains, since the exact MIC of 4-EOTC was not determined, it was assumed to be 512 µg/mL for the calculation of the FICI of the OTC and 4-EOTC combination. Using this assumed value ensures that the real FICI would not be higher than the value determined here; therefore, the interpretation does not overestimate the effect of the combination. In fact, if the actual MIC (4-EOTC) is greater than 512 µg/mL, the corresponding FICI would be lower than the one calculated in this study. To our knowledge, the present study demonstrates for the first time that the combination of OTC with its degradation product 4-EOTC, at a concentration equal to the initial MIC of OTC, enhances the antibacterial activity of OTC, yielding a FICI <0.5 and thus indicating synergy. With a similar objective, another study was conducted to compare the activity of colistin alone and in combination with four of its degradation products (26). Authors demonstrated that the presence of these four degradation products enhanced the antibacterial activity of colistin against some *E. coli* strains (26), although the FICI was not calculated.

The antibacterial activity of 4-EOTC should be further investigated in future studies to characterize its spectrum of activity across a broad range of bacterial species, including Gram-positive bacteria. Given that OTC is derived from *Streptomyces rimosus*, 4-EOTC could serve as a starting point for the development of a new generation of semi-synthetic tetracyclines, in which part of the molecule would be produced by *Streptomyces*, while the remaining portion could be derived from chemical synthesis.

In the present study, using the conjugation assay, the plasmid carrying the *tetA* gene from a donor APEC strain was successfully transferred to a tetracycline-susceptible recipient, *E. coli* K-12. After conjugation, the MICs of OTC and 4-EOTC were determined against the transconjugant strain, both exceeding 256 µg/mL. These results confirm that *tetA* was located on a mobile plasmid and may be the principal determinant conferring resistance to OTC and 4-EOTC in the tested strain. However, as a limitation of our study, the conjugation assay was performed using only one APEC strain, which harbors a single tetracycline resistance gene, *tet*A. Therefore, future studies are necessary to validate the activity against 4-EOTC of *tetA* and other tetracycline resistance genes across different APEC strains, as well as in other bacterial species.

In the present study, using the simulated poultry gizzard and HPLC-MS/MS analysis, no degradation of OTC was observed after 2 h of incubation at 42°C in an acidic medium. Moreover, no change in the antibacterial activity of OTC was observed after 2 h of this simulation. The choice of a 2 h simulated gizzard condition was justified by the residence time of a drug or a feed ingredient in this biological compartment of chickens (49). This lack of degradation of OTC, in this first compartment of the poultry digestive tract, may be explained by the chemical structure of OTC, which lacks peptide bonds susceptible to pepsin activity (50), in contrast to colistin, which is rich in such peptide bonds and therefore rapidly degrades under simulated gastric conditions (26). Hence, the OTC metabolites that were identified, in poultry, in eggs, plasma (51), liver, muscles, feathers (52), claws (53), and droppings (54), could be the result of OTC degradation in other segments of the gastrointestinal tract or during the first hepatic pass. Consequently, additional studies are needed to characterize *in vivo*, following oral administration, the presence of OTC metabolites in the bloodstream, as well as in other poultry organs (liver, kidneys), and to determine their half-lives compared to the parent compound, OTC.

### Conclusion

This study provides the first comprehensive assessment of OTC degradation products, revealing that 4-EOTC retains antibacterial activity against APEC strains and exhibits synergistic effects with OTC, while α-apo-OTC and β-apo-OTC are inactive. In the present study, the *tetA* gene was shown to confer resistance to both OTC and 4-EOTC, and OTC remains stable under simulated poultry gizzard conditions. These findings highlight that 4-EOTC is a promising lead for next-generation tetracyclines and underscore the importance of considering degradation products in AMR dynamics and management.

## Data Availability

The authors declare that the main data supporting the findings of this work are available within the article and its supplemental materials files or from the corresponding author upon reasonable request.

## SUPPLEMENTAL MATERIAL

Table S1 spectrum.03768-25-s0001.docx Combinatory effect of oxytetracycline (OTC) and α-apo-OTC.

Table S2 spectrum.03768-25-s0002.docx Combinatory effect of oxytetracycline (OTC) and β-apo-OTC.

Table S3 spectrum.03768-25-s0003.docx Predicted genetic characterization of reconstructed plasmids.

ASM does not own the copyrights to Supplemental Material that may be linked to, or accessed through, an article. The authors have granted ASM a non-exclusive, world-wide license to publish the Supplemental Material files. Please contact the corresponding author directly for reuse.
