# Supplementary material for: First in vitro characterization of three oxytetracycline degradation products against avian pathogenic Escherichia coli isolates: antibacterial activity, formation in gizzard, and resistance gene profiling
Source: Microbiol Spectr. 2026 Jul 6;14(8):e03768-25. doi: 10.1128/spectrum.03768-25 (PMC13436374; doi:10.1128/spectrum.03768-25)
Supplement: Supplementary file 2 [file spectrum.03768-25-s0002.docx]

**Table S2.** Combinatory effect of oxytetracycline (OTC) and β-apo-OTC against tetracycline-resistant avian pathogenic *Escherichia coli* (APEC) strains (n=7) determined by checkboard method.

|  | | | **OTC with β-apo-OTC at different concentrations (µg/mL)** | | | | | | | | | | | | | | | |
| --- | --- | --- | --- | --- | --- | --- | --- | --- | --- | --- | --- | --- | --- | --- | --- | --- | --- | --- |
| APEC strains | Initial MIC (OTC) | MIC (β-apoOTC) alone* | 256 | | 128 | | 64 | | 32 | | 16 | | 8 | | 4 | | 2 | |
|  |  |  | MIC (OTC) | FICI | MIC (OTC) | FICI | MIC (OTC) | FICI | MIC (OTC) | FICI | MIC (OTC) | FICI | MIC (OTC) | FICI | MIC (OTC) | FICI | MIC (OTC) | FICI |
| ECL15813** | 512 | >256 | >256 | 2 | >256 | 2 | >256 | 2 | >256 | 2 | >256 | 2 | >256 | 2 | >256 | 2 | >256 | 2 |
| ECL25364** | 512 | >256 | >256 | 2 | >256 | 2 | >256 | 2 | >256 | 2 | >256 | 2 | >256 | 2 | >256 | 2 | >256 | 2 |
| ECL20552 | 256 | >256 | 256 | 2 | 256 | 2 | 256 | 2 | 256 | 2 | 256 | 2 | 256 | 2 | 256 | 2 | 256 | 2 |
| ECL24206 | 256 | >256 | 256 | 2 | 256 | 2 | 256 | 2 | 256 | 2 | 256 | 2 | 256 | 2 | 256 | 2 | 256 | 2 |
| ECL25618 | 256 | >256 | 256 | 2 | 256 | 2 | 256 | 2 | 256 | 2 | 256 | 2 | 256 | 2 | 256 | 2 | 256 | 2 |
| ECL24026 | 256 | >256 | 256 | 2 | 256 | 2 | 256 | 2 | 256 | 2 | 256 | 2 | 256 | 2 | 256 | 2 | 256 | 2 |
| ECL21752 | 256 | >256 | 256 | 2 | 256 | 2 | 256 | 2 | 256 | 2 | 256 | 2 | 256 | 2 | 256 | 2 | 256 | 2 |

OTC: oxytetracycline; FICI: fractional inhibitory concentration index; MIC: minimal inhibitory concentration; Blue columns: highlight neutral effect (1<FICI<4). All assays were conducted in triplicate.

*For these strains, the MIC of β-apo-OTC was considered to be 512.

**For ECL15813 and ECL25364 strains, the MIC of OTC in presence of β-apo-OTC was considered to be 512.
