# Supplementary material for: First in vitro characterization of three oxytetracycline degradation products against avian pathogenic Escherichia coli isolates: antibacterial activity, formation in gizzard, and resistance gene profiling
Source: Microbiol Spectr. 2026 Jul 6;14(8):e03768-25. doi: 10.1128/spectrum.03768-25 (PMC13436374; doi:10.1128/spectrum.03768-25)
Supplement: Supplementary file 3 [file spectrum.03768-25-s0003.docx]

**Table S3.** Predicted genetic characterization of reconstructed plasmids and associated tetracycline resistance genes in tetracycline-resistant avian pathogenic *Escherichia coli* (APEC) strains (n=7) using MOB-suite, as previously described [30].

| Strain ID | Plasmid cluster | Nearest replicon(s) | Replicon type accession(s) | Relaxase type | Predicted mobility | Gene symbol | Sequence name | % Identity to reference sequence (*tet*) | Accession of closest sequence (*tet)* |
| --- | --- | --- | --- | --- | --- | --- | --- | --- | --- |
| ECL24206 | AA176 | IncFIB, IncFIC,rep_cluster_2244 | 000095__NZ_CP014273_00094,AY458016,CP033091_00053 | not determined | no predicted mobility* | *tet(A)* | tetracycline efflux MFS transporter Tet(A) | 100 | WP_000804064.1 |
| ECL24026 | NA | chromosomal | NA | NA | NA | *tet(A)* | tetracycline efflux MFS transporter Tet(A) | 100 | WP_000804064.1 |
| ECL20552 | AA474 | IncI-gamma/K1 | KT186369 | MOBP | no predicted mobility* | *tet(A)* | tetracycline efflux MFS transporter Tet(A) | 100 | WP_000804064.1 |
| ECL21752 | AA474 | IncI-gamma/K1 | KT186369 | MOBF | no predicted mobility* | *tet(A)* | tetracycline efflux MFS transporter Tet(A) | 100 | WP_000804064.1 |
| ECL25364 | AA176 | IncFIB,IncFIC,rep_cluster_2244 | 000095__NZ_CP014273_00094,AY458016,CP033091_00053 | MOBF | conjugative | *tet(B)* | tetracycline efflux MFS transporter Tet(B) | 100 | WP_001089068.1 |
| ECL15813 | AA474 | IncI-gamma/K1 | KT186369 | MOBP | conjugative | *tet(A)* | tetracycline efflux MFS transporter Tet(A) | 100 | WP_000804064.1 |
| ECL25618 | AA176 | IncFIC, rep_cluster_2244 | AY458016,CP033091_00053 | MOBF | conjugative | *tet(A)* | tetracycline efflux MFS transporter Tet(A) | 100 | WP_000804064.1 |
